# Chaetocin induces apoptosis in human melanoma cells through the generation of reactive oxygen species and the intrinsic mitochondrial pathway, and exerts its anti-tumor activity *in vivo*

**DOI:** 10.1371/journal.pone.0175950

**Published:** 2017-04-18

**Authors:** Xinming Han, Yan Han, Yongsheng Zheng, Qiang Sun, Tao Ma, Junyi Zhang, Lianji Xu

**Affiliations:** 1 Department of Plastic and Reconstructive Surgery, Chinese PLA General Hospital, Beijing, China; 2 Medical Cosmetic Center, Beijing Tongren Hospital, Capital Medical University, Beijing, China; Institute of Biochemistry and Biotechnology, TAIWAN

## Abstract

Chaetocin is a small-molecule natural product produced by *Chaetomium* species fungi, and it has a potent anti-proliferative pharmacological activity on various cancer cells. However, the effect of chaetocin on anti-melanoma pharmacological role has not been investigated. Therefore, in this study, we explored the effect of chaetocin on cell proliferation in the human melanoma Sk-Mel-28 and A375 cells and the growth of tumor xenografts in nude mice. The results indicated that chaetocin treatment significantly suppressed cell proliferation and induced apoptosis in the Sk-Mel-28 and A375 cells in a dose- and time-dependent manner. Furthermore, chaetocin treatment resulted in an increased level of cellular reactive oxygen species (ROS), and pre-incubation of cells with N-acetylcysteine (NAC) significantly abrogated chaetocin-induced apoptosis in the melanoma cells. A significant reduction of mitochondrial membrane potential and the release of cytochrome c were observed after chaetocin treatment. Additionally, chaetocin treatment significantly up-regulated the protein levels of Bax, cleaved caspase-9/-3, simultaneously down-regulated the protein levels of Bcl-2, procaspase-9/-3, and activated caspase-9/-3 activity in the melanoma cells. The *in vivo* data demonstrated that chaetocin treatment significantly inhibited the growth of melanoma tumor xenografts in nude mice, which was closely associated with apoptosis induction, a reduced level of PCNA (proliferating cell nuclear antigen) expression, and activation of capase-9/-3 in tumor xenografts. These are the first data to demonstrate that chaetocin exerts a proapoptotic activity on human melanoma cells through ROS generation and the intrinsic mitochondrial pathway. Therefore, chaetocin might represent an effective candidate for melanoma chemotherapy.

## 1. Introduction

Melanoma is one of the most aggressive forms of skin cancers with a high frequency of metastasis and with very poor prognosis in the metastatic stage [1]. Although melanoma represents 4% of dermatologic cancers, it is responsible for 80% of skin cancer deaths because of its aggression, metastasis and drug-resistance [2]. Efficient treatment requires early diagnosis. If patients were early diagnosed with primary melanoma, surgical resection is the best choice for most of them to reduce mortality [3]. However, a 5-year survival rate in metastatic melanoma is still under 15–20% of patients [4]. Therefore, novel therapeutic strategies that inhibit melanoma growth and progression need to be developed for improving the survival of patients with melanomas [5].

Chaetocin is a small-molecule natural product produced by *Chaetomium* species fungi [6,7], and its chemical structure belongs to diketoepiperazines, and was described in 1970 [8]. However, its effects on cellular processes were studied only in the two past decades. It has been reported that chaetocin has a potent and selective *in vitro* and *in vivo* anti-myeloma activity as it can induce cellular oxidative stress [9]. Additionally, chaetocin was then found to have a strong inhibitory effect on a broad range of cancer cells including human chronic myelogenous leukemia cells [10], glioma cells [11], non-small cell lung cancer cells [12], and renal cell carcinoma cells [13]. Recently, Bae et al. found that chaetocin could inhibit melanogenesis in B16F10 mouse melanoma cells via suppressing the protein level of microphthalmia-associated transcription factor (MITF) and followed by activation of the extracellular signal-regulated kinases (ERK) signaling pathway [14]. However, the pharmacological action of chaetocin on human melanoma cells remains unclear. In this study, we investigated the inhibitory effects of chaetocin on the growth of human melanoma SK-Mel-28 and A375 cells and tumor xenografts in nude mice, and explored its underlying molecular mechanisms for chaetocin-induced apoptosis *in vitro* and *in vivo*.

## 2. Materials and methods

### 2.1 Cell culture and reagents

Human melanoma cell lines, Sk-Mel-28, A375, IGR37, LU-1205 and MV3 were purchased from Shanghai Cell Bank of Chinese Academy of Sciences, and they were cultured in RPMI-1640 medium supplemented with 10% fetal bovine serum (FBS) (Gibco, USA), penicillin (100 IU/ml), and streptomycin (100 μg/ml) in a humidified incubator with 5% CO_2_ at 37°C. Human primary melanocytes were obtained Kanglang Company (Shanghai, China) and cultivated in melanocytes growth with 10% FBS. 3-[4, 5-dimethylthiazol-2-yl]-2, 5-diphenyltetrazolium bromide (MTT) and chaetocin were purchased from Sigma-Aldrich Corp. (St. Louis, MO). Chaetocin was dissolved in dimethyl sulfoxide (DMSO) to prepare a 50 mM stock solution which was diluted to the final concentration with culture medium. The final concentration of DMSO was kept under 0.1% throughout the following studies, and showed no effect on cell morphology and proliferation in this study. The primary antibodies recognizing Bax, Bcl-2, procaspase-9/-3, cleaved caspase-9/-3, cytochrome c, PCNA, Nrf2, SOD2, catalase, and β-actin were purchased from Abcam Company (Cambridge, UK).

### 2.2 Cell viability assay

Cell viability was evaluated by MTT assay. Briefly, Sk-Mel-28, A375, IGR37, LU-1205, MV3, normal melanocytes were seeded in 96-well plates at 5 × 10^3^ cells/well containing 0.1 ml growth medium and incubated overnight, followed by treatment with indicated concentrations for 24, 48, and 72 h. 20μl of MTT solution were then added to each well and incubated for 4 h at 37°C. Formazan crystals were dissolved in 150 μl of DMSO and quantitated spectrophotometrically at a wavelength of 570nm with a micro-plate reader to obtain absorbance values. Cell viability was expressed as percentage change compared to the control. The IC_50_ value was determined by curve fitting of the sigmoidal dose-response curve. The experiments were independently performed for three times.

### 2.3 Apoptosis analysis

Apoptotic cells were determined using flow cytometry with CellQuest software (BD Biosciences, Franklin Lakes, NJ) after staining with an Annexin V-FITC/PI kit (Life Technologies, Carlsbad, CA). Briefly, the cells were treated with 0, 5 and 10 μM chaetocin for 24 h or treated with 10 μM chaetocin for 24, 48 and 72 h. Then, the cells were collected and incubated with Annexin V-FITC/PI for 10 min in the dark, and analyzed with flow cytometry. Annexin V-FITC^+^/PI^−^ cells represented early apoptotic cells, whereas V-FITC^+^/PI^+^ cells were considered to be in the late stage of apoptosis and necrotic cells.

### 2.4 Detection of intracellular levels of ROS

The levels of intracellular ROS were determined as described previously [15], with some modifications. Briefly, the cells were treated with various concentrations of chaetocin for 12, 24 and 48 h, followed by removal of culture medium. According to the instructions of a ROS assay kit (BioVision, San Francisco, CA), 100 μl of 2, 7-dichlorodihydrofluorescein diacetate (DCFH-DA) solution were gently mixed with 2.5 × 10^4^ cells, and added into 96-well plates for incubation of 1 h in the dark. The fluorescence intensity of each well was determined with a fluorescence plate reader at Ex/Em. = 488/525 nm. The experiments were independently performed at least three times.

### 2.5 Determination of N-acetyl cysteine effects on chaetocin-induced apoptosis

Sk-Mel-28 and A375 cells were seeded in 6-well plates. After incubation overnight, the cells were pre-treated with 4 mM N-acetyl cysteine (NAC) for 2 h, followed by incubation with 10 μM chaetocin for 24 h, and cellular apoptosis was determined as described above.

### 2.6 Measurement of mitochondrial membrane potential (Δψm)

Sk-Mel-28 and A375 cells were grown for 24 h in a 12-well plate (1 × 10^5^ cells/ well) and incubated with 0, 5 and 10 μM chaetocin for 12 h in a incubator, followed by washing with PBS and analyzed with JC-1 mitochondrial membrane potential assay kit (Biotium company, Hayward, CA)following the manufacturer’s instruction. Briefly, 1 ml of culture medium containing 75 μl of JC-1 staining solution was added into each well for incubation of 30 min at 37°C. The cells were then analyzed with FACS calibur (BD Bioscience, Franklin Lakes, NJ) to detect JC-1 fluorescence at excitation wavelength of 488 nm and emission at 590 nm and 529 nm for J-aggregate (red fluorescence) and J-monomeric (green fluorescence) forms, respectively. The mitochondrial membrane potential was obtained through the ratio of red fluorescence to green fluorescence [16]. Higher value of the ratio indicates better mitochondrial functioning.

### 2.7 Western blot analysis

Melanoma cells Sk-Mel-28 and A375 were treated with 0, 5 and 10 μM chaetocin for 48 h. The cells were then collected and lysed in lysis buffer [0.5M Tris-HCl (pH7.4), 10mM ethylenediaminetetraacetate (EDTA), 1mM sodium orthovanadate, 1.5M NaCl, 1mM phenylmethylsulfonyl fluoride (PMSF), 2.5% deoxycholic acid, 0.02%NaN3, 1% NP-40, a protease inhibitor cocktail set (Roche, Germany)] for 30 min on ice. The lysates were separated by centrifugation at 4°Cat 12,000×g for 10 min. Supernatant soluble proteins were collected, and determined with a BCA protein assay kit (Beyotime, Haimen, China) to obtain the total protein concentration. Equal amounts of protein were separated on 10% SDS-polyacrylamide gel (SDS-PAGE), and then transferred to polyvinylidenedifluoride (PVDF) membranes, which were saturated with 5% skim milk in 1×TBST (Tris-buffered saline and 1% Tween 20), and then incubated with primary antibodies overnight at 4°C. After washing three times with TBST, the membranes were incubated with goat anti-rabbit IgG-HRP secondary antibody for 1 h, followed by detection using an enhanced chemiluminescence reagent and Odyssey Infrared Imaging System (LI-COR Inc., NE).

### 2.8 Determination of cytochrome c release

Cytochrome c release was detected as described previously [17]. In brief, Sk-Mel-28 and A375 cells were incubated without or with 5 and 10 μM chaetocin for 48 h. Then, the cytoplasm and mitochondria fractions were separated using an ApoAlert Cell Fractionation kit (Mountain View, CA, USA) according to the manufacturer’s instructions. Subsequently, cytosolic protein concentration was determined, and then subjected to western blot analysis.

### 2.9 Caspase-9 and caspase-3 activity assay

Caspase-9 and caspase-3 activity was measured as described previously [18]. Briefly, 5 × 10^4^ cells were seeded in each well of 96-well plates, and incubated for 24 h, followed by treatment with 0, 5 and 10 μM chaetocin for 48 h. 100 μl of Caspase-Glo 9/3 reagent (Promega, Madison, WI) was then added for the incubation of 2 h at room temperature. Luciferase activity in the cells was measured using a microplate luminometer (Promega).

### 2.10 Inhibitory effects of chaetocin on tumor xenograft growth in nude mice

Female athymic nude mice (4–5 weeks of age, about 20 g in weight) were purchased from Beijing Laboratory Animal Research Center (Beijing, China), and housed at the Animal Center of Chinese PLA General Hospital. Mice were maintained under a 12-h light and 12-h dark cycle in an air-conditioned room with specific pathogen-free conditions, and were provided rodent chow and water *ad lilitum*. The animal tests were accordance with the guidelines of Animal Care and Use Committee (ACUC) of Chinese PLA General Hospital, and the animal protocol used in this study was reviewed and approved by the ACUC of Chinese PLA General Hospital. 1 × 10^6^ cells (Sk-Mel-28 or A375 cells) were injected subcutaneously into the right hind flanks of nude mice. After cell inoculation for 12 days, the tumor volume reached 100 ± 10 mm^3^. Mice were randomly divided into different treatment groups with six mice each group, followed by a daily intraperitoneal injection with chaetocin at a dose of 2 mg/kg for 20 days based on our preliminary data. The control groups were treated with the same amount of saline. Body weight of each mouse was recorded every two days. Tumor volume was measured by length (L) and width (W) every four days with an external caliper, and tumor size was calculated as described previously [19]: tumor volume = π/6(L × W)^3/2^. All of the mice were sacrificed at the end of 20 days treatment, and the tumor tissues were removed and weighted. A port of tumor tissues were embedded in paraffin and used for immunohistochemical analysis, and the others were used to prepare tumor tissue lysates for western blot analysis of active caspase-9/-3, Bax and Bcl-2 protein expression levels.

### 2.11 Immunohistochemistry and TUNEL assay

Immunohistochemical staining for proliferating cell nuclear antigen (PCNA) expression was performed to investigate cell proliferation in the tumor tissues. The excised tumor tissues from nude mice were embedded in paraffin, and sectioned into 5 μm thick slices, which were then deparaffinized and rehydrated, followed by antigen retrieval as described previously [20]. The sections were incubated with 3% bovine serum albumin for 30 min to block the non-specific binding sites, and then incubated with anti-PCNA antibody overnight at 4°C, followed by incubation with biotinylated secondary antibody for 30 min. The sections were then incubated with avidin-biotin-peroxidase complex (Vector Laboratoies Ltd., Peterborough, UK) for 1 h and with 3, 3-diaminobenzidine (Sigma-Aldrich) for 20 min for visualization, followed by counterstaining with hematoxylin. The terminal deoxynucleotidyl transferase-mediated dUTP nick end labeling (TUNEL) assay was performed using a TUNEL in situ apoptosis detection kit (FITC) (Qcbio Ltd., Shanghai, China) according to the manufacturer’s instructions. The slices were finally observed and captured under a microscope with a digital camera (Olympus, Tokyo, Japan).

### 2.12 Statistical analysis

Data were presented as the mean ±SEM. Statistical analysis was performed by one-way ANOVA with post-hoc comparisons with the Turkey test using SPSS software (version 11.0). The value of *P*< 0.05 was considered statistically significant.

## 3. Results

### 3.1 Chaetocin inhibits cell viability of melanoma cells

**Fig 1:**
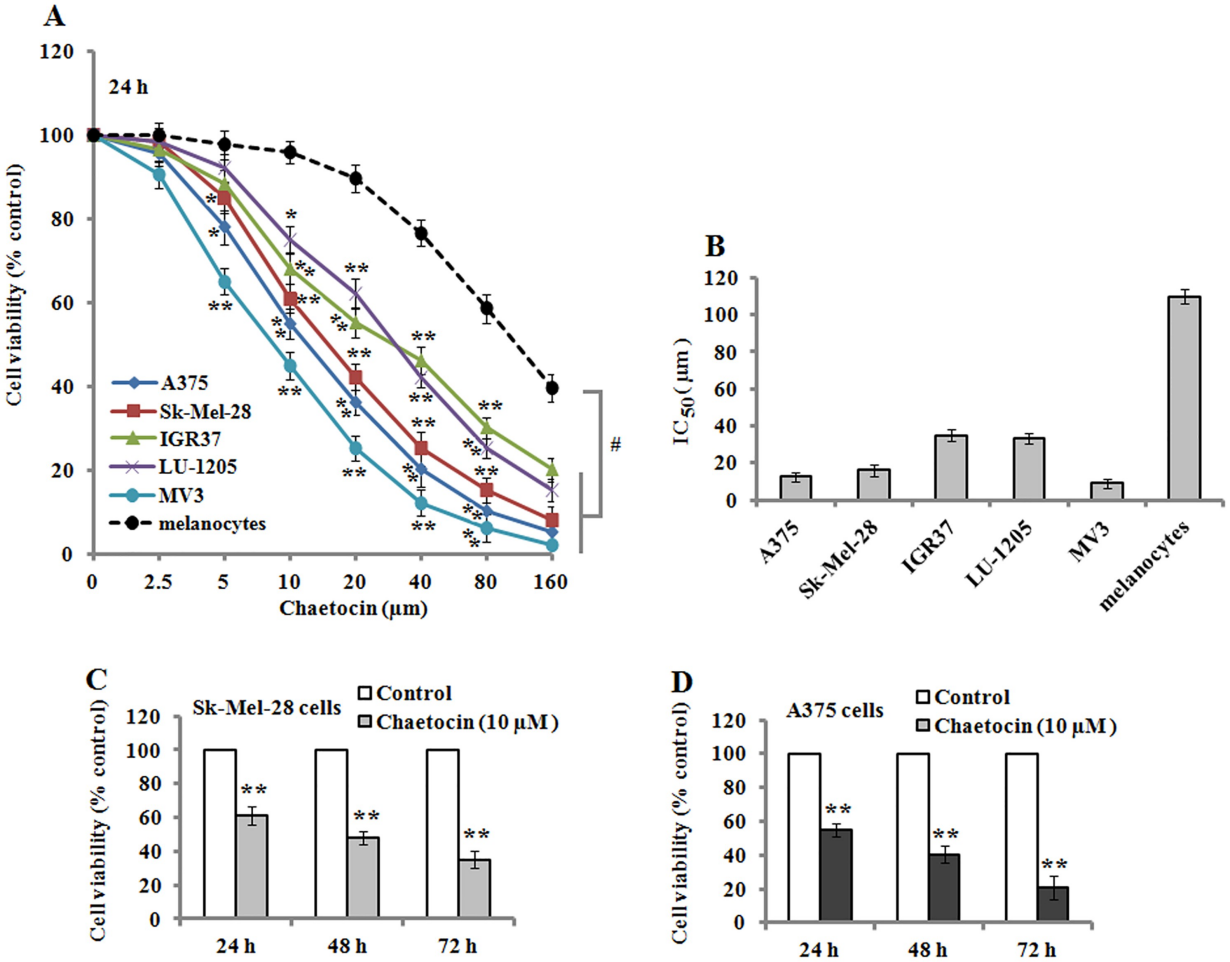
Chaetocin inhibits cell viability in melanoma cells. Cells (A375, Sk-Mel-28, IGR37, LU-1205, MV3, and normal melanocytes) were treated with different doses of chaetocin for 24 h, and cell viability was determined by MTT assay. (A) Chaetocin suppressed cell proliferation in a dose-dependent manner. (B) The IC_50_ was determined when various cells were treated by chaetocin for 24 h. (C-D) Sk-Mel-28 and A375 cells were treated with 10 μM chaetocin for 24, 48 and 72 h, and displayed a time-dependent inhibitory effect on cell viability. Data represented as means ± SEM (n = 3). *P< 0.05 and **P< 0.01, with the untreated controls. ^#^*P*< 0.05, compared with normal melanocytes.

To investigate the effects of chaetocin treatment on cell viability, human melanoma cells, A375, Sk-Mel-28, IGR37, LU-1205, MV3, and normal human primary melanocytes were treated with various concentrations of chaetocin (0, 2.5, 5, 10, 20, 40, 80, and 160μM) for 24 h. The results of MTT assay demonstrated that chaetocin strongly decreased the viability of melanoma cells (A375, Sk-Mel-28, IGR37, LU-1205, and MV3) in a concentration-dependent manner (5–160μM), and all these melanoma cells were more susceptible to chaetocin than normal human primary melanocytes (Fig 1A). The IC_50_ values in the melanoma cells and melanocytes were 12.55± 2.31 μM(A375), 16.02± 3.21μM (Sk-Mel-28), 35.01± 3.15μM (IGR37), 33.49± 2.89μM (LU-1205), 9.01± 2.34μM(MV3), and 110.02± 3.91μM (melanocytes) after 24 h treatment (Fig 1B). Additionally, we used 10 μM chaetocin to treated Sk-Mel-28 and A375 cells for 24, 48 and 72 h, cell viability was significantly inhibited when compared with the untreated control cells (Fig 1C and 1D), which demonstrated the inhibitory effect of chaetocin on melanoma cell viability in a time-dependent manner.

### 3.2 Chaetocin induces apoptosis in human melanoma cells

**Fig 2:**
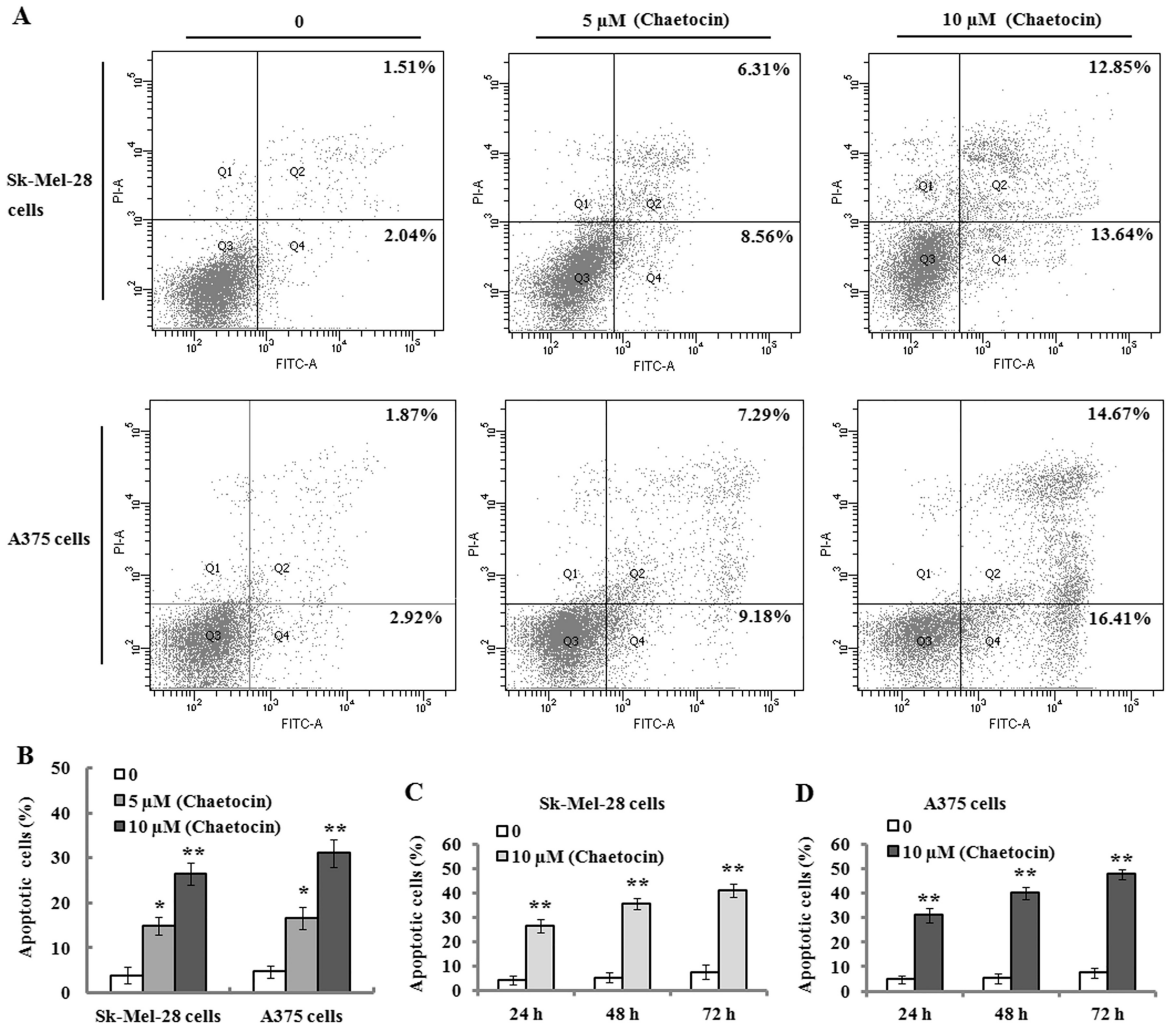
Chaetocin induces apoptosis in human melanoma cells. Apoptosis was detected by Annexin V-FITC/PI using flow cytometry. (A) The representative plots of flow cytometry for chaetocin-induced apoptosis in Sk-Mel-28 and A375 cells which were treated with 0, 5 and 10 μM chaetocin for 24 h. (B) The cells were treated with indicated concentrations of chaetocin for 24 h, and demonstrated an increasing apoptotic rate in a dose-dependent manner. (C-D) Sk-Mel-28 (C) and A375 (D) cells were treated without or with 10 μM chaetocin for 24, 48 and 72 h, and apoptotic cells were determined with flow cytometry. Data represented as means ± SEM (n = 3). *P< 0.05 and **P< 0.01, compared with the controls.

To investigate whether chaetocin induces apoptosis in Sk-Mel-28 and A375 cells, flow cytometric analysis was performed. The results indicated that chaetocin (5 and 10 μM) treatment on the Sk-Mel-28 and A375 cells for 24 h resulted in a significant increase of apoptotic cells (Annexin V-FITC^+^/PI^−^ and Annexin V-FITC^+^/PI^+^ cells), and demonstrated an apoptosis-induced effect in a concentration-dependent manner (Fig 2A and 2B). Additionally, 10 μM chaetocin treatment on these cells for 24, 48 and 72 h produced a gradual increase of apoptotic cells (Fig 2C and 2D), which displayed that chaetocin could induce apoptosis in Sk-Mel-28 and A375 cells in a time-dependent manner.

### 3.3 Chaetocin causes mitochondrial membrane potential (Δψm)loss and increases ROS production

**Fig 3:**
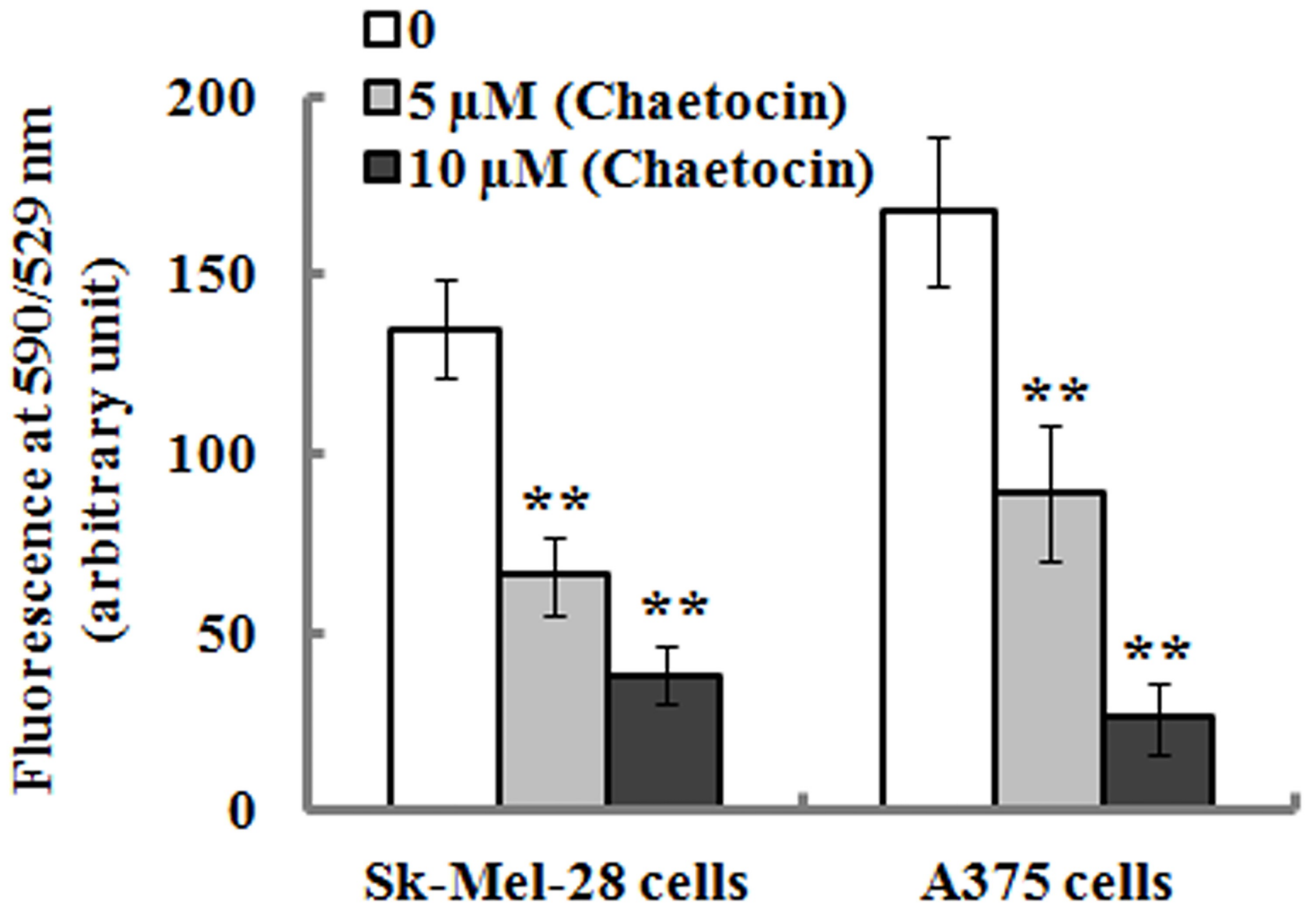
Chaetocin reduces mitochondrial membrane potential (Δψm). SK-Mel-28 and A375 cells were incubated in the presence or absence of chaetocin for 12 h, and mitochondrial membrane potential was analyzed as described in Materials and methods. Chaetocin significantly decreased mitochondrial membrane potential of Sk-Mel-28 and A375 cells. Data represented as means ± SEM (n = 3). **P< 0.01, compared with the controls.

Cellular Δψm was evaluated with JC-1 dye as well as flow cytometry analysis. A decrease of cellular Δψm is indicated by a reduction in red/green fluorescence intensity ratio. As shown in Fig 3, chaetocin treatment significantly resulted in Δψm loss in the Sk-Mel-28 and A375 cells after 12 h incubation, which suggests that chaetocin-induced apoptosis was associated with mitochondrial dysfunction.

**Fig 4:**
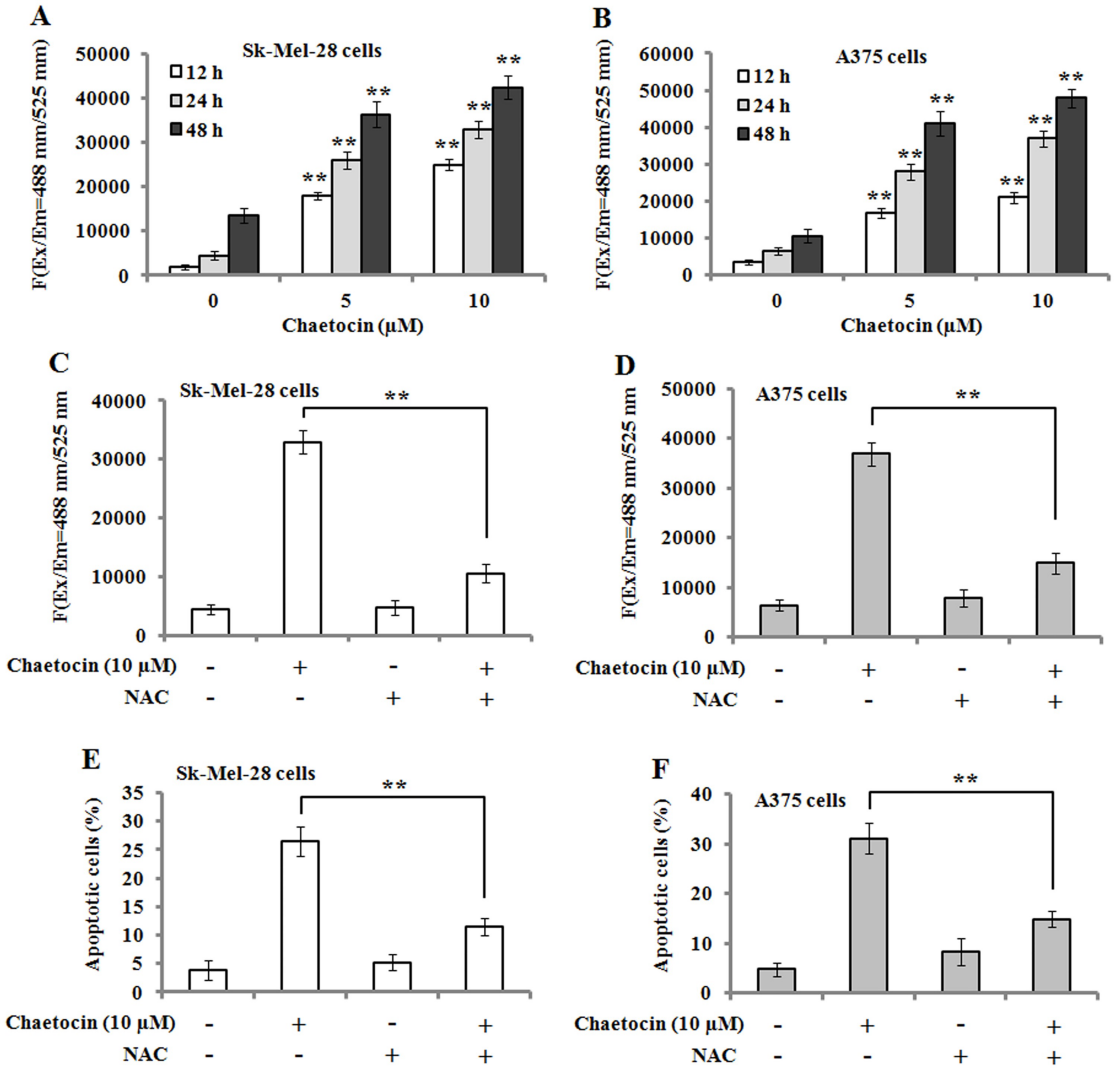
Chaetocin treatment produces ROS and N-Acetyl Cysteine (NAC) reduces chaetocin-induced apoptotic effects in human melanoma cells. (A-B): Chaetocin treatment resulted in ROS generation in Sk-Mel-28 (A) and A375 (B) cells, as detected by a fluorescence plate reader. Data represented as means ± SEM (n = 3). **P< 0.01, compared with controls. (C-D): NAC pre-treatment reduced chaetocin-induced ROS generation in Sk-Mel-28 (C) and A375 (D) cells. (E-F) NAC pre-treatment attenuated chaetocin-induced apoptosis in Sk-Mel-28 (E) and A375 (F) cells. Data represented as means ± SEM (n = 3). **P< 0.01.

It is well-known that increased generation of ROS can trigger a caspase of events resulting in apoptotic occurrence. Therefore, the level of ROS was measured in Sk-Mel-28 and A375 cells that were treated with various concentrations of chaetocin for 12, 24 and 48 h. The results showed that chaetocin significantly elevated the level of ROS in the cells in a time- and concentration- dependent manner (Fig 4A and 4B). However, pre-treatment of Sk-Mel-28 and A375 cells with 4 mM N-acetyl cysteine (NAC, ROS scavenger) showed a significantly decreased level of ROS compared to that of chaetocin-treated cells (Fig 4C and 4D). Further investigation exhibited that NAC pre-treatment attenuated chaetocin-induced apoptosis in Sk-Mel-28 and A375 cells (Fig 4E and 4F). These results suggest that ROS was significantly involved in chaetocin-induced apoptosis.

### 3.4 Chaetocin alters the expression of oxidative stress pathway related genes

**Fig 5:**
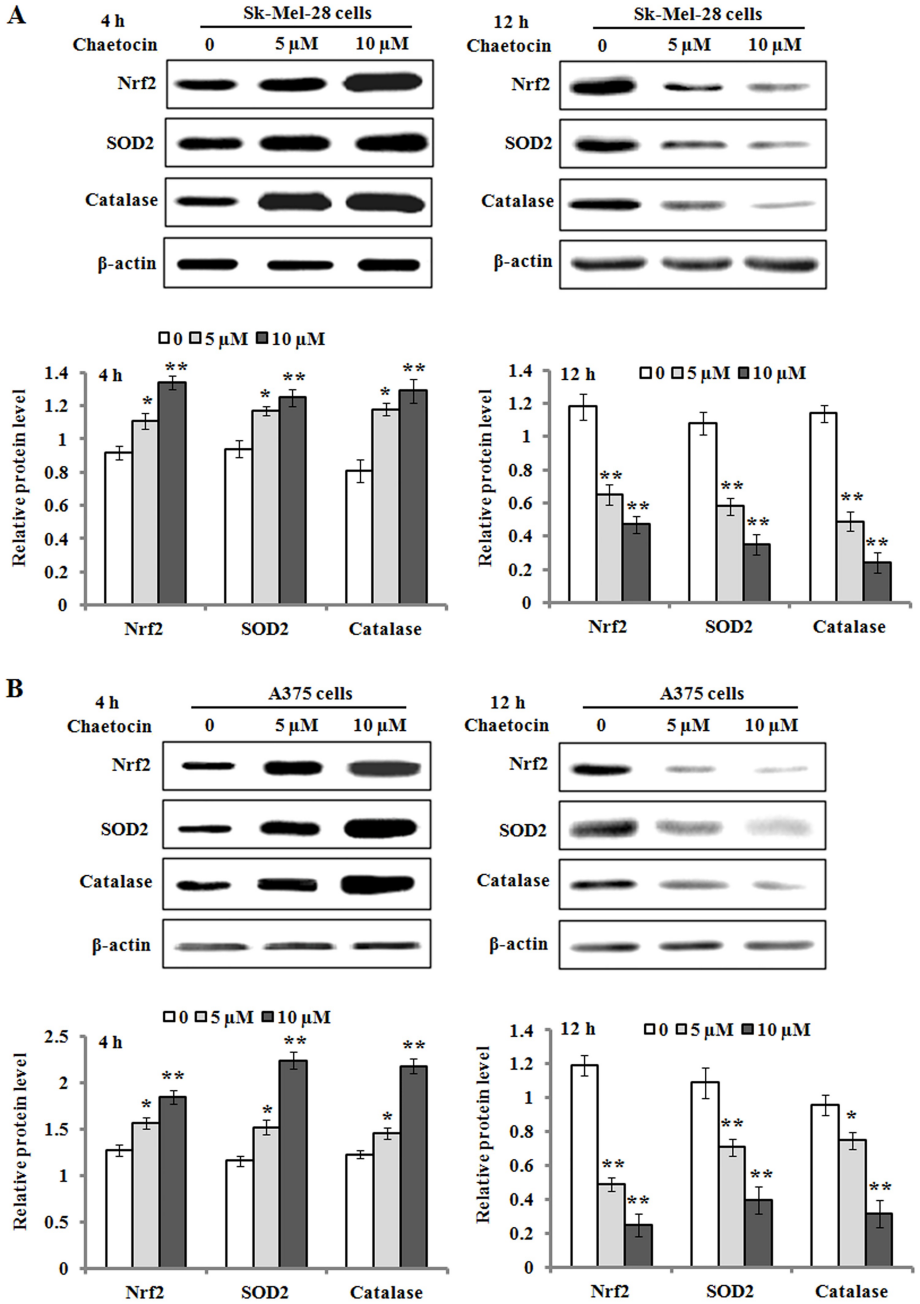
The effects of chaetocin treatment on the expression of Nrf2, SOD2 and catalase. (A-B) The cells were treated with 0, 5 and 10 μM chaetocin for 4 and 12 h, and the protein levels of Nrf2, SOD2, and catalase from Sk-Mel-28 (A) and A375 cells (B) were evaluated Western blotting. The results of Western blotting were scanned and analyzed by NIH image J 3.0. The protein levels of Nrf2, SOD2 and catalase were quantified by densitometric ratio of targeted protein/β-actin. Data represented as means ± SEM (n = 3). *P< 0.05 and **P< 0.01, compared with the controls. SOD2: Superoxide dismutase 2.

Nrf2 is a sensor for oxidative stress and regulates the activation of defensive genes [21]. Additionally, superoxide dismutase 2(SOD2) and catalase play an important role in redox balance to scavenge the elevating level of ROS [22]. The Sk-Mel-28 and A375 cells treated with 5 and 10 μM of chaetocin for 4 h significantly upregulated the protein expression levels of Nrf2, SOD2and catalase compared to the control (Fig 5). However, with the extension of chaetocin treatment to 12 h, the expression levels of Nrf2, SOD2 and catalase were markedly downregulated in the cells.

### 3.5 Chaetocin induces cytochrome c release and activates the apoptotic pathway

**Fig 6:**
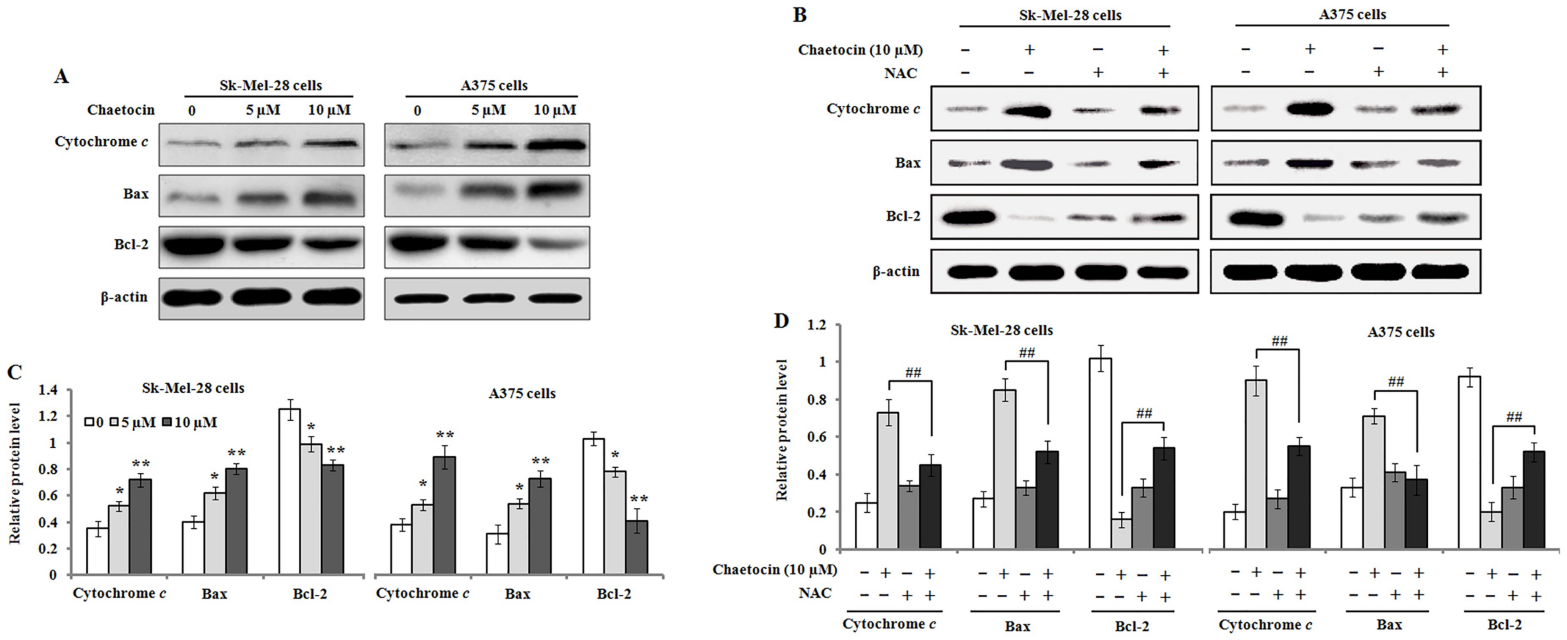
The effects of chaetocin treatment on cytochrome c release and Bax and Bcl-2 expression in melanoma cells. (A) The cells were treated with 0, 5 and 10 μM chaetocin for 48 h, and the protein expression of cytochrome *c*, Bax and Bcl-2 in Sk-Mel-28 and A375 cells was analyzed with Western blotting. (B) Pre-treatment with N-acetyl cysteine (NAC) counteracted the chaetocin-mediated effects on the protein expression of cytochrome *c*, Bax and Bcl-2 in the cells. (C-D) The protein levels of cytochrome c, Bax and Bcl-2 were quantified by densitometric ratio of targeted protein/β-actin. Data represented as means ± SEM (n = 3). *P< 0.05 and **P< 0.01, compared with the controls; ^##^*P*< 0.01, compared with 10μM chaetocin.

To further study the effect of chaetocin on mitochondrial integrity that is closely related with the balance between anti-apoptotic and pro-apoptotic protein mediators. We determined the release of cytochrome c from mitochondria in the Sk-Mel-28 and A375 cells that were incubated with 0, 5 and 10 μM chaetocin for 48 h. The results of western blot analysis showed that a gradually increased level of cytosolic cytochrome c was observed with the increasing concentrations of chaetocin, which implied that chaetocin treatment resulted in cytochrome c release from cellular mitochondrial. The results also showed that chaetocin treatment markedly elevated the level of pro-apoptotic protein Bax, and concurrently significantly lowered the level of anti-apoptotic protein Bcl-2 in the Sk-Mel-28 and A375 cells exposed to 5 and 10 μM chaetocin for 48 h. But NAC pre-treatment attenuated the release of cytochrome c, decreased Bax expression, and up-regulated Bcl-2 protein level (Fig 6).

**Fig 7:**
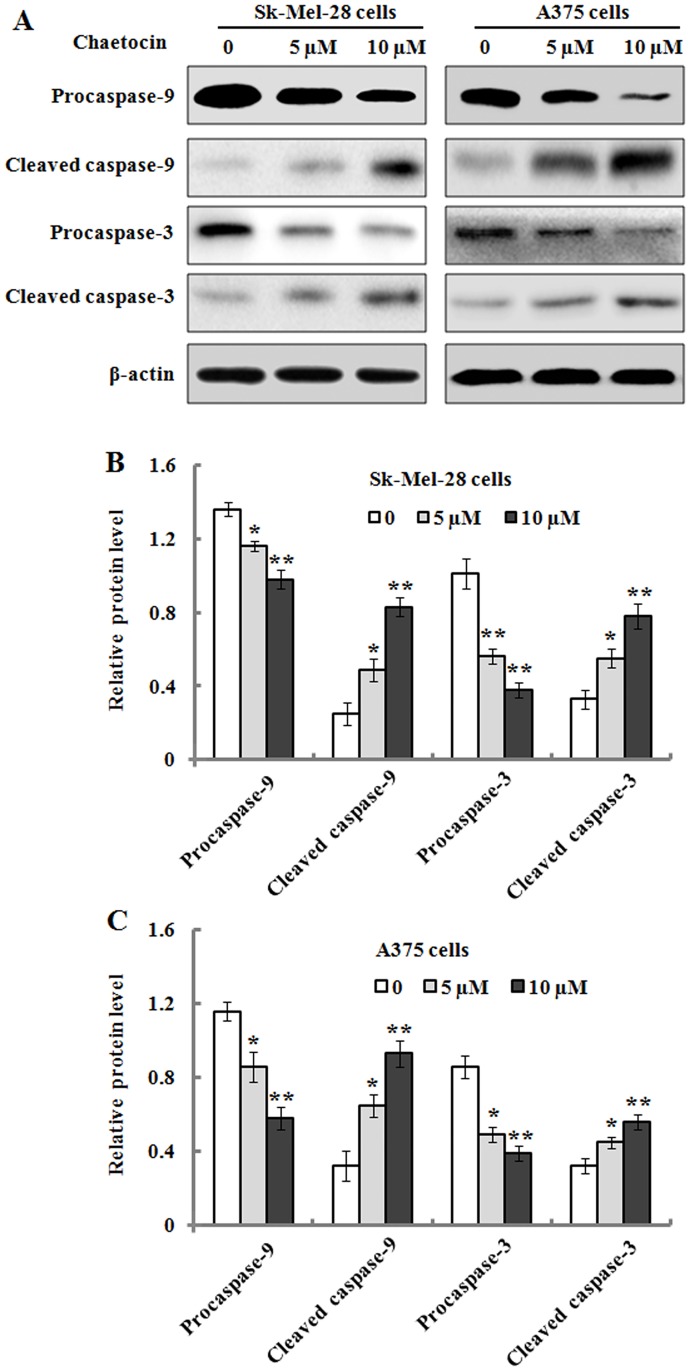
The effects of chaetocin treatment on the expression level of caspase-9/-3. Sk-Mel-28 and A375 cells were treated with 0, 5 and 10 μM chaetocin for 48 h, and cellular lysates were then prepared and subjected to SDS-PAGE analysis. (A): Representative western blot images for procaspase-9, cleaved caspase-9, procaspase-3, and cleaved caspase-3. (B-C): The relative protein levels of procaspase-9, cleaved caspase-9, procaspase-3, and cleaved caspase-3 in Sk-Mel-28 (B) and A375 (C) cells were quantified by densitometry. The data are represented as means ± SEM (n = 3). *P< 0.05 and **P< 0.01, compared with the controls.

**Fig 8:**
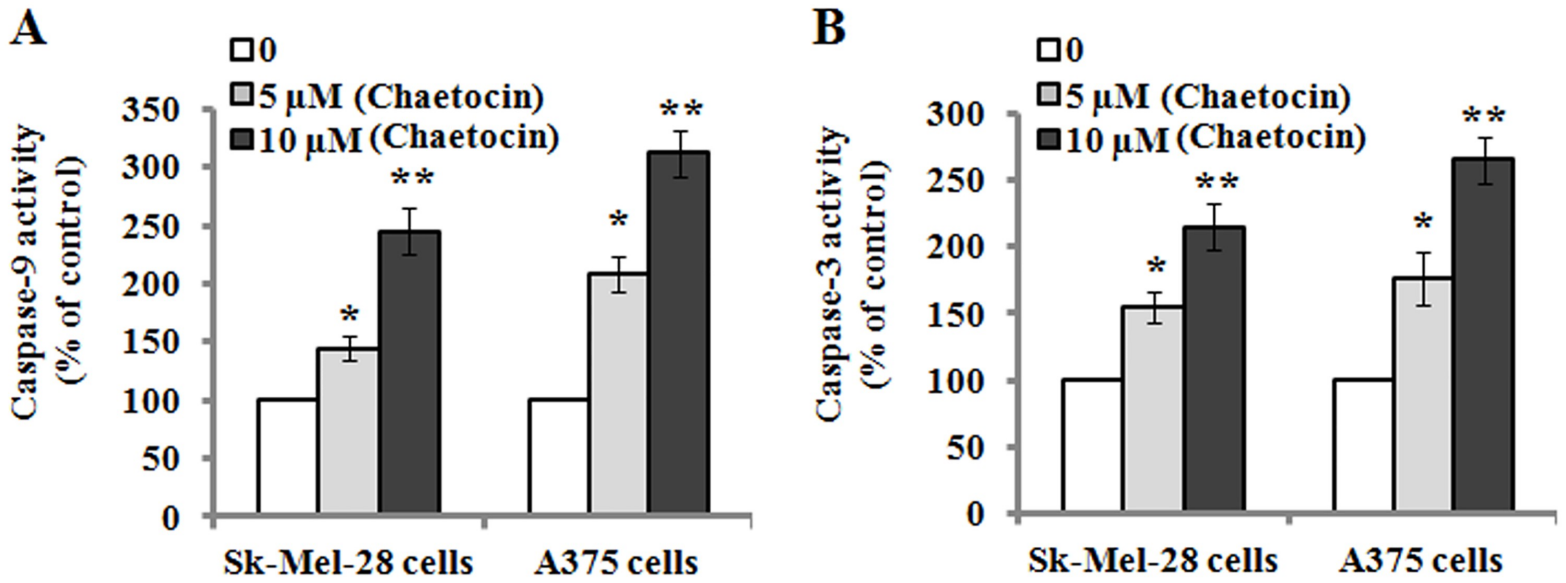
Chaetocin treatment elevates the activities of caspase-9 and caspase-3. (A-B): Sk-Mel-28 and A375 cells were treated with 0, 5 and 10 μM chaetocin for 48 h, and their caspase-9 and caspase-3 activities were measured as described in Materials and methods. Data represented as means ± SEM (n = 3). *P< 0.05 and **P< 0.01, compared with the controls.

It has been reported that caspase family has a critical role in the initiation and execution of apoptotic events [23]. Accordingly, we measured whether or not enzymatic procaspase-9/-3 was activated during chaetocin-induced apoptosis in Sk-Mel-28 and A375 cells. As shown in Fig 7, the results exhibited that intact procaspase-9/-3 was gradually down-regulated in the cells after exposure to the increasing concentrations of chaetocin for 48 h; whereas the proteolytic cleaved forms of caspase-9/-3 was found to be up-regulated. These results indicated that chaetocin treatment could induce caspase-9/-3 activation in Sk-Mel-28 and A375 cells, which was further confirmed by their activity analysis. As shown in Fig 8, caspase-9/-3 activity was significantly elevated in the chaetocin-treated Sk-Mel-28 and A375 cells in a concentration-dependent manner when compared to the untreated cells, which suggested that caspase-dependent pathway was involved in chaetocin-induced apoptotic death in the Sk-Mel-28 and A375 cells.

### 3.6 Chaetocin suppresses the growth of tumor xenograft in nude mice

**Fig 9:**
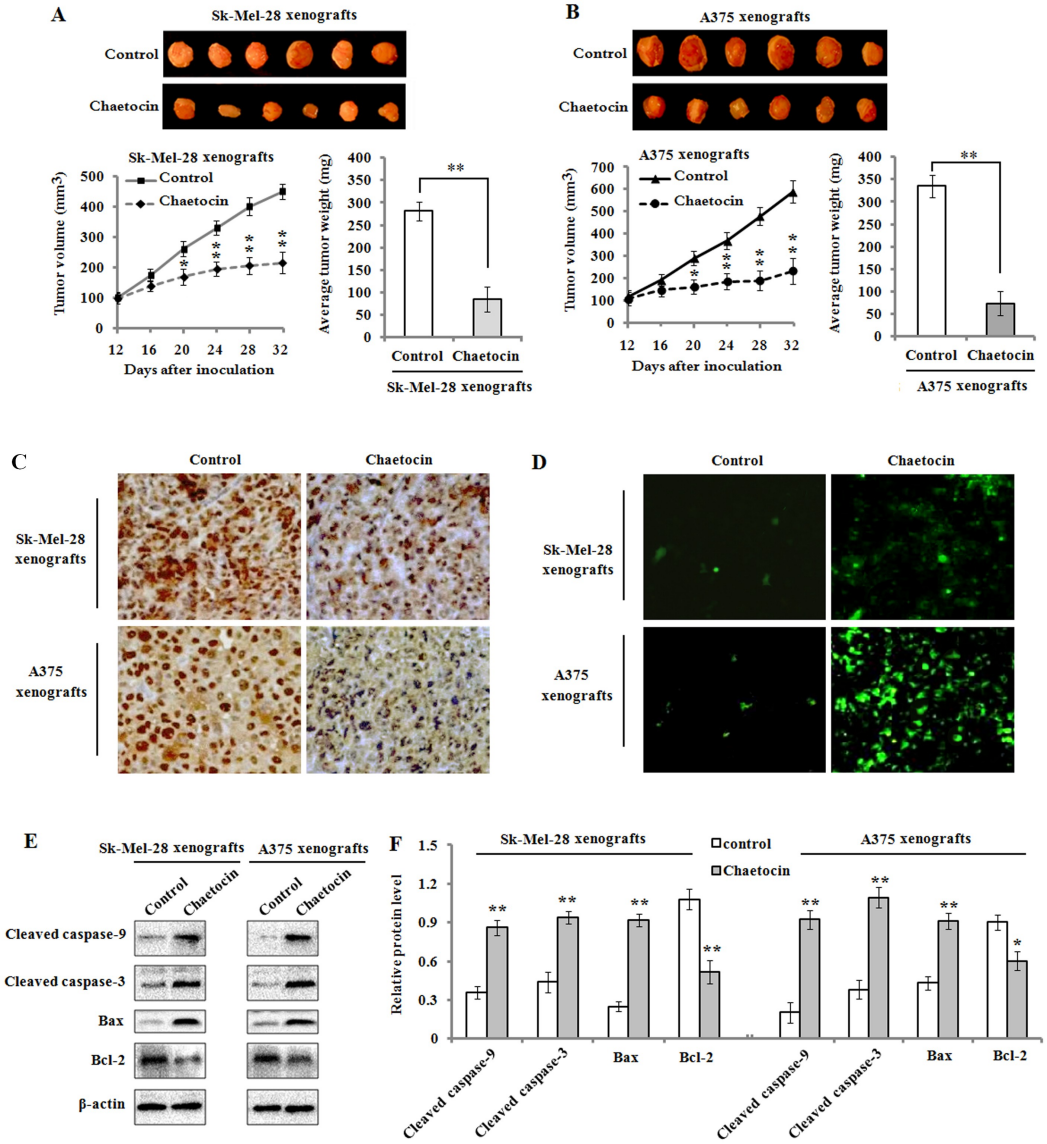
Chaetocin inhibits tumor growth in xenografts. Mice xenografted with Sk-Mel-28 and A375 cells were intraperitoneally injected with chaetocin (2 mg/kg/day) for 20 days when the tumor volume reached 100 ± 10 mm^3^ (on 12^th^ days of cell inoculations). (A-B): Tumor volume was assessed every 4 days, and average tumor weight was determined after the mice were sacrificed at the end of treatment. *P< 0.05 and **P< 0.01 vs controls, n = 6/group. (C): Immunohistochemical staining was used to detect PCNA expression (dark brown) in the xenograft sections. Original magnification: 200 ×. (D): TUNEL assay was used to evaluate cellular apoptosis (green) in Sk-Mel-28 and A375 xenografts from control (saline) and chaetocin treated mice. Original magnification: 200 ×. (E): Western blot analysis of the protein expression levels of active caspase-9/-3 (cleaved caspase-9/-3), Bax and Bcl-2 in the tumor lysates of control and chaetocin-treated mice. (F): Quantification of cleaved caspase-9/-3, Bax and Bcl-2 protein levels in tumor the lysates of control and chaetocin-treated mice. *P< 0.05 and **P< 0.01 vs controls, n = 6/group.

Next, we detected the effect of chaetocin administration on the growth of tumor xenograft in the nude mice that were inoculated with Sk-Mel-28 or A375 cells. At 12 days after cell inoculation, mice were intraperitoneally injected with chaetocin (2 mg/kg daily) for up to 20 days, while controls received a same amount of saline. The results showed that chaetocin treatment strongly inhibited the growth of tumor xenograft of Sk-Mel-28 and A375 cells (Fig 9A and 9B). Tumor volume in the chaetocin-treated mice was markedly suppressed compared to the control mice. At the end of the experiments (day 32), a significant reduction of average tumor weight was observed in chaetocin-treated mice when compare to the control mice, whereas chaetocin administration did not significantly affect animal body weight measured every two days throughout the experimental period (data not shown). On the other hand, we analyzed the anti-proliferative effect of chaetocin on the melanoma tumor xenografts using immunohistochemical staining for the detection of PCNA expression. The results showed that PCNA-positive cells (dark brown) in the tumor xenograft tissues from chaetocin-treated mice were markedly decreased (Fig 9C), which suggested that chaetocin treatment could effectively suppress cell proliferation in tumor xenografts. Additionally, to investigate the role of chaetocin on cellular apoptosis *in vivo*, TUNEL assay was performed. The results demonstrated that TUNEL positive cells (green) were obviously increased in the tumor xenograft tissues from chaetocin-treated mice, whereas only few TUNEL positive cells were observed in the control mice (Fig 9D). To further investigate whether intrinsic pathways of apoptosis observed *in vitro* also functioned in vivo, western blot analysis was applied to detect the expression levels of active caspase-9/-3 (cleaved caspase-9/-3), Bax and Bcl-2 in tumors. The results exhibited that active caspase-9/-3 were significantly upregulated in the chaetocin treated group compared with control group in Sk-Mel-28 and A375 xenografts. Additionally, an increased level of pro-apoptotic Bax and a decreased level of anti-apoptotic Bcl-2 protein were obviously found in the tumor tissue lysates from chaetocin-treated mice (Fig 9E and 9F).

## 4. Discussion

Malignant melanoma is the most deadly form of skin cancer, with strongly invasive capacity and high mortality rates [24]. It was reported that the incidence of melanoma had rising in the past years, and there were approximately 232,000 new cases happened each year worldwide [25]. However, effective systemic therapies for this disease are currently still short because of drug resistance and serious side effects. Accordingly, novel agents need to be explored to overcome the current limitation of therapeutic strategies. In the present study, we demonstrated that chaetocin, a thiodioxopiperazine natural product previously found to have anticancer effects [9], had a potent activity against melanoma cells and only minor cytotoxicity was observed in normal melanocytes when low concentration(less than 10μM) of chaetocin was applied. It was found that chaetocin strongly induced apoptosis in the human melanoma Sk-Mel-28 and A375 cells through ROS generation and activation of intrinsic mitochondrial pathway. Furthermore, chaetocin treatment significantly attenuated tumor growth in the melanoma cells xenograft model of nude mice. To our knowledge, this is the first report that chaetocin-induced apoptosis in Sk-Mel-28 and A375 melanoma cells, which implies that chaetocin represents a promising agent for melanoma treatment in future.

Chaetocin is a natural product produced by *Chaetomium* species fungi, and was found to have a potent *in vitro* and *in vivo* anti-myeloma activity attributed to induction of ROS imposed by inhibition of thioredoxin reductase [26]. In the present study, chaetocin significantly suppressed cell proliferation and induced apoptosis in the melanoma cells in a dose- and time-dependent manner, as shown in the Sk-Mel-28 and A375 cells. Previous studies have indicated that oxidative stress is an important regulator of apoptosis in tumor cells, and apoptotic occurrence can be induced by chemotherapeutic agents through elevation in ROS generation and perturbation of redox homeostasis [11, 27]. In this study, an elevated level of ROS in the Sk-Mel-28 and A375 cells were observed following chaetocin treatment. Pre-incubation of cells with NAC antioxidant clearly abrogated the elevated level of ROS in chaetocin-treated cells, protected the cells from chaetocin-induced apoptosis. These findings are in agreement with previous reports indicating chaetocin-induced apoptosis is blocked by antioxidant treatment [11, 28]. Additionally, we detected the expression level of genes such as Nrf2, SOD2 and catalase involved in oxidative stress-induced apoptotic following treatment with chaetocin. The expression of Nrf2, SOD2 and catalase was upregulated when the cells were treated with chaetocin for 4 h. But with the extension of chaetocin treatment to 12 h, a sharp decrease of Nrf2, SOD2 and catalase expression was observed. It is postulated that the antioxidant defense system in the Sk-Mel-28 and A375 cells was triggered in response to increase cellular oxidative stress generated by chaetocin treatment of 2 h, which resulted in the upregulation of Nrf2, SOD2 and catalase expression to scavenge the chaetocin-mediated elevating level of ROS. Nonetheless, the ROS level induced by chaetocin was very high and surpassed the antioxidant capacity of antioxidants including SOD2 and catalase when the cells were incubated with chaetocin for 12 h, which led to the apoptotic occurrence in Sk-Mel-28 and A375 cells and subsequent downregulation of Nrf2, SOD2 and catalase expression. These results indicate that a high level of ROS production at least partly accounts for chaetocin-induced apoptosis in Sk-Mel-28 and A375 cells.

Currently, apoptosis induction is considered as a predominant therapeutic approach for various bioactive agents and natural products. It has been reported that there are two major proapoptotic pathways in apoptosis control [29]. Cell death receptors and their ligands, such as CD95L/FasL, tumor necrosis factor-α (TNF-α) or TNF related apoptosis-inducing ligand (TRAIL), generally involve in extrinsic pathway [30]. On the other hand, intrinsic pathway, called as mitochondrial pathway, are initiated by various kinds of cellular dysregulation, which leads to the depolarization of mitochondrial membrane potential and the efflux of cytochrome c from mitochondria to cytosol, where the initiator caspase-9 is activated and thus results in the activation of other caspase members, such as caspase-3 and caspase-7 [31]. In the present study, we found that chaetocin treatment resulted in the loss of mitochondrial membrane potential and the release of cytochrome c from mitochondria in the Sk-Mel-28 and A375 cells. Previous studies have indicated that Bcl-2 family including anti-apoptotic proteins (Bcl-2, Bcl-x_L_, Bcl-w and Mcl-1) and pro-apoptotic proteins (Bax and Bak) play an important role in the intrinsic apoptotic pathway by regulation of mitochondria integrity [32]. Anti-apoptotic proteins such as Bcl-2 and Bcl-xL can prevent the release of apoptogenic proteins from mitochondria. On the contrary, pro-apoptotic proteins such as Bax and Bak can induce mitochondrial membrane permeabilization and promote the release of pro-apoptotic factors including cytochrome c [33,34]. In agreement with its effects, we found that the level of Bax protein was up-regulated and that of Bcl-2 was down-regulated in Sk-Mel-28 and A375 cells when treated with chaetocin. Therefore, the ratio of Bax/Bcl-2 was significantly increased. These results suggest that increased Bax and reduced Bcl-2 expression contribute to chaetocin-mediated apoptosis in the Sk-Mel-28 and A375 cells. Caspases, which are cysteine-containing aspartate-specific proteases, play critical roles in the initiation and execution of apoptosis [35]. Based on their functions in apoptosis, caspases have been divided into two groups including initiator caspases such as caspase-8 and caspase-9, and effector caspases such as caspase-3 [36]. The extrinsic pathway is activated by receptor-mediated caspase-8 while the intrinsic pathway is activated by cytochrome c-mediated caspase-9 [37]. In the present study, our findings showed that procaspase-9 and procaspase-3 were cleaved and down-regulated, and the cleaved fragments (caspase-9/-3) were found to be increased after chaetocin treatment in the Sk-Mel-28 and A375 cells, which suggests that procaspase-9 and procaspase-3 were activated in the cells. The results of Caspase-Glo 9/3 assay further confirmed that the activity of caspase-9 and caspase-3 was significantly elevated following chaetocin treatment. These data indicated that chaetocin-induced apoptosis was regulated by caspase-dependent pathway.

Based on the *in vitro* results, we sought to confirm the inhibitory effect of chaetocin against melanoma growth using an *in vivo* tumor xenograft model in nude mice. Consistent with the *in vitro* studies, we found that chaetocin significantly attenuated tumor growth without any apparent sign of toxicity in the Sk-Mel-28 and A375 cell xenografts, reduced tumor volume at different times of treatment, and decreased the average tumor weight at the end of the treatments. Additionally, inhibition of PCNA expression and apoptotic cell death of tumor cells in the tumor xenograft tissues were observed in the chaetocin-treated mice. The increased Bax and decreased Bcl-2 in the tumor lysates from chaetocin-treated mice were also observed with the activation of caspase-9/-3, which is closely associated with chaetocin-induced apoptosis.

## 5. Conclusions

In summary, our results indicate that chaetocin inhibits cell proliferation, and induces apoptosis in the human melanoma Sk-Mel-28 and A375 cells through ROS generation and intrinsic apoptotic pathway. Additionally, our results also indicate that chaetocin attenuates tumor growth in the Sk-Mel-28 and A375 cell xenograft model of nude mice, and displays a strong anti-tumor activity. Therefore, these findings suggest that chaetocin has the potential to be developed as a new agent for melanoma treatment.
